# The impact of tick-borne pathogen infection in Indian bovines is determined by host type but not the genotype of *Theileria annulata*

**DOI:** 10.1016/j.meegid.2019.103972

**Published:** 2019-11

**Authors:** S.D. Larcombe, S.W. Kolte, G. Ponnudurai, N. Kurkure, S. Magar, R. Velusamy, N. Rani, B. Rubinibala, B. Rekha, A. Alagesan, W. Weir, B.R. Shiels

**Affiliations:** a Institute of Biodiversity, Animal Health and Comparative Medicine, University of Glasgow, UK; b Department of Veterinary Parasitology, Faculty of Tamil Nadu Veterinary and Animal Sciences University, Chennai, India; c Department of Veterinary Parasitology, Nagpur Veterinary College, Maharashtra Animal and Fisheries Sciences University, Nagpur, India; d Department of Veterinary Parasitology, Veterinary College and Research Institute, Namakkal, India; e School of Veterinary Medicine, College of Medical, Veterinary and Life Sciences, University of Glasgow, Scotland G61 1QH, UK

**Keywords:** *Theileria annulata*, *Anaplasma*, Apicomplexa, Microsatellites, Breed resistance, Cattle

## Abstract

Tick-borne pathogens (TBP) are a major source of production loss and a welfare concern in livestock across the globe. Consequently, there is a trade-off between keeping animals that are tolerant to TBP infection, but are less productive than more susceptible breeds. *Theileria annulata* is a major TBP of bovines, with different host types (i.e. exotic and native cattle breeds, and buffalo) displaying demonstrable differences in clinical susceptibility to infection. However, the extent to which these differences are driven by genetic/physiological differences between hosts, or by different parasite populations/genotypes preferentially establishing infection in different host breeds and species is unclear. In this study, three different bovine host types in India were blood sampled to test for the presence of various TBP, including *Theileria annulata*, to determine whether native cattle (*Bos indicus* breeds), crossbreed cattle (*Bos taurus* x *Bos indicus* breeds) or water buffalo (*Bubalus bubalis*) differ in the physiological consequences of infection. Population genetic analyses of *T. annulata* isolated from the three different host types was also performed, using a panel of mini- and micro-satellite markers, to test for sub-structuring of the parasite population among host types. We discovered that compared to other host types, “carrier” crossbreed cattle showed a higher level of haematological pathology when infected with *T. annulata*. Despite this finding, we found no evidence for differences in the genotypes of *T. annulata* infecting different host types, although buffalo appeared to harbour fewer mixed parasite genotype infections, indicating they are not the major reservoir of parasite diversity. The apparent tolerance/resistance of native breed cattle and buffalo to the impacts of *T. annulata* infection is thus most likely to be driven by host genotype, rather than differences in the parasite population. Our results suggest that an improved understanding of the genetic factors that underpin disease resistance could help to ameliorate future economic loss due to TBP or tropical theileriosis.

## Highlights

• *Theileria annulata* infection has different consequences in crossbreed cattle, native cattle and water buffalo in India
• Crossbreed *Bos taurus* x *Bos indicus* cattle showed reductions in haematological parameters when infected with *T. annulata*
• We found no evidence for infection-mediated physiological change in native cattle breeds or water buffalo
• We found no sub-structuring of the *T. annulata* population between different host types using microsatellite analysis
• Buffalo were not a major reservoir of genetically diverse *T. annulata* infecting cattle, contrasting the epidemiology of *T. parva*

## 1 Introduction

Tick-borne pathogens (TBP) have major welfare and economic implications across the globe. Costs associated with cattle ticks and the pathogens they transmit are estimated to be as high as 18.7 billion USD per year (de Castro, 1997). A large proportion of this cost is attributed to livestock mortality and loss of productivity in dairy and beef cattle suffering from anaplasmosis (primarily caused by *Anaplasma marginale*), theileriosis (primarily caused by *Theileria annulata* and *T. parva*) and babesiosis (primarily caused by *Babesia bigemina* and *B. bovis*). A general and long-standing control measure against tick-borne disease (TBD) is the application of acaricides (Graham and Hourrigan, 1997). Acaricides, however, are associated with several drawbacks including reduced efficacy due to tick resistance, sensitivity of hosts to their application and a detrimental impact on the environment (George et al., 2004). Alternative approaches are therefore vital for the sustainable improvement of productivity and animal health. One strategy is to promote rearing of cattle breeds, including crossbreed, with increased genetic resistance to ticks and the diseases they transmit in an attempt to reduce economic loss and improve productivity.

The role of host species and breed in determining resistance to disease caused by various bovine TBP is generally well supported. For example, mortality caused by *T. parva* is generally low in native breed (*Bos indicus*) cattle in endemic areas, but as high as 100% in non-endemic *B. taurus* or *B. taurus* x *indicus* crossbreeds (Glass et al., 2005). For *T. annulata*, some native *B. indicus* breeds in endemic areas are also considered resistant to clinical disease, and research has shown differences in expression of a number of bovine genes between parasite-infected cell lines from resistant and susceptible hosts (Jensen et al., 2008). Thus, with the exception of anaplasmosis caused by *Anaplasma marginale*, resistance to (or tolerance of) TBP is much greater in native *B. indicus* cattle breeds and buffalo than in imported *B. taurus* cattle breeds (Bock et al., 1997, Bock et al., 1999). Pure *B. indicus* breeds lack the productivity traits of *B. taurus*, however, and this has led to the implementation of a major *B. indicus* x *B. taurus* crossbreeding programme in India in an effort to select for more productive/resistant cattle, primarily to reduce losses due to tropical theileriosis caused by *T. annulata*. It is well recognised that animals that do not succumb to acute disease, i.e. those that might be considered to show resistance, still suffer detrimental consequences of infection. Both sub-acute and chronic disease occurs in some animals, including crossbreeds, and though animals can recover, convalescence is often protracted and some individuals will eventually succumb (Brown et al., 1990). Importantly, a further group of sub-clinically infected animals may also be present, i.e. carriers. These animals have no visible clinical signs and may possess only a very low parasitaemia that is often undetectable by microscopy. Recently, it has been shown that such carrier infections may represent a hidden but important economic cost, particularly in crossbreeds (Kolte et al., 2017), and that subclinical carrier infections are in fact associated with subtle pathological changes that impact productivity (Perera et al., 2014). It has also been demonstrated that *T. annulata* carrier infections can be more economically important than clinical infections in endemic regions of Tunisia (Gharbi et al., 2011). Despite this, most field studies remain focussed on the most seriously diseased individuals, and in many cases where carriers are considered, groups of different host types are not located in the same habitat and may therefore be exposed to different epidemiological conditions. Recent studies on tick-borne pathogens in India have found that, even on a farm-by-farm scale, differences in location can have a profound impact on disease epidemiology (Kolte et al., 2017; Ponnudurai et al., 2017). To fully understand the role of host type on susceptibility to parasite infection, consideration should also be given to the interaction between host and parasite genotype, i.e. host genotypes may be tolerant or more susceptible to a subset of parasite genotypes that establish infection; and/or some parasite genotypes may show greater virulence when encountering a subset of host genotypes. Overall, little is currently known about the genetic diversity of *Theileria* parasites in India and, more specifically, whether different sub-populations of parasite genotypes may preferentially infect the three major bovine host types. In *T. parva*, it has been demonstrated that cattle harbour far fewer parasite genotypes than African Cape buffalo (*Syncerus caffer*), suggesting the more disease tolerant/resistant buffalo act as a reservoir of infections for other hosts with only a subset of the total *T. parva* population capable of establishing infection in more susceptible cattle (Bishop et al., 1994; Collins and Allsopp, 1999; Geysen et al., 2004; Oura et al., 2003). Whether this is also true for *T. annulata* has not been established.

In this study we used data from co-grazed populations of native breed cattle, crossbreed cattle and water buffalo from two different regions of India to answer three key questions: (1) does host type influence the impact of subclinical infection on physiological traits linked to production; (2) does host type influence the progression of infection to the clinical stage and the severity of clinical signs; and (3) is the parasite population sub-structured among different host types? Answering these questions will be important to help shape future strategies to improve the economic output of livestock farming in the face of continuing challenge by TBP in endemic regions.

## 2 Materials and methods

### 2.1 Sample collection and diagnostic testing

Cattle were blood sampled in two regions of India where disease caused by TBP is considered to be a major problem, Maharashtra State and Tamil Nadu. Data was collected from bovines in each region representing the three different host types: native breed cattle, crossbreed cattle and water buffalo. In every case we sampled only where animals were co-grazed together in the same habitat, or were in close enough proximity that tick-borne transmission was extremely likely (i.e. grazing and drafting animals on the same farm). The use of co-grazed animals allowed us to control for the geographical site of sampling. Further details on the locations and characteristics of these study areas, as well as local patterns of TBP prevalence at the time of this study can be found in Kolte et al. (2017) and Ponnudurai et al. (2017). For every animal from each region, a polymerase chain reaction (PCR) was previously used to diagnose infection with *T. annulata*, *T. orientalis*, *B. bovis*, *B. bigemina* and *Anaplasma* spp. Details of the PCR protocols are described in Kolte et al. (2017). Thereafter, different aspects of our analyses were conducted on the subsets of samples from the two different regions as follows.

### 2.2 Impacts of subclinical TBP infection

To assess whether host type influences the outcome of infection at a subclinical but physiologically detectable level, we analysed samples from 319 animals from Tamil Nadu that were not displaying overt clinical signs associated with infection. Data on factors influencing prevalence and distribution of different tick species and TBP from these animals was previously published (Ponnudurai et al., 2017), which showed that host age and breed type significantly impacted the prevalence of TBP, though impacts on the host were not assessed. In the present study, we wished to assess how these infections with TBP, principally *T. annulata* and *Anaplasma* spp., impact health-related metrics from a subset of animals that showed no clinical signs at time of sampling, and had no history of clinical signs for at least 6 months prior to (and after) blood samples being taken. Therefore, the animals were classified as subclinical carriers at the time of sampling, although we cannot exclude, entirely that the animals had never displayed signs of TBD previously. From each of the 319 animals, we analysed a blood sample to record packed cell volume (PCV), haemoglobin (Hb) concentration, red blood cell (RBC) counts and white blood cell (WBC) counts, using the biochemistry analyser VetScan VS2 (Abaxis Veterinary Diagnostics, Union City, CA 94587, USA). These measurements have previously been linked to infection-mediated changes in productivity of dairy cattle (Perera et al., 2014) and so were assessed in the present study to provide evidence of important physiological changes in animals not experiencing severe clinical disease.

### 2.3 Impact of host type and TBP on progression to clinical infection

To assess the likelihood of infections progressing to the clinical stage, an analysis was performed using samples from Maharashtra state (see Kolte et al., 2017 for details of the study area). Animals from Tamil Nadu were not used for this comparison, as too few clinical cases were reported to our field veterinarians during the study period. Field veterinarians monitored the health of animals in Maharashtra state, with data on the presence of clinical signs being recorded. Clinical disease was indicated if at least one of the following signs was noted: lymphadenopathy, diarrhoea, ptyalism, debilitation and pyrexia. Blood samples were identified from animals showing these clinical signs, which are associated with acute TBP (*n* = 80). These 80 clinical cases were compared with data from a further 153 non-clinical (carrier) cases from identical sampling regions (as reported in Kolte et al., 2017) to assess enrichment for clinical cases among the different host types, or TBP cases.

### 2.4 Population genetics of *T. annulata*

We wished to assess whether genetic sub-structuring of the principal and most important TBP in both regions, *T annulata,* could be detected among the different host types. If present, this could explain previously reported differences in parasite prevalence, or the physiological consequences of infection (in terms of progression to clinical disease or deterioration in haematological parameters) reported here. For population genetic analysis, samples from ten farms across both Maharashtra state (*n* = 121) and Tamil Nadu (*n* = 20) were utilised. All of the samples were from *T. annulata*-positive blood samples from the three major host types.

A panel of ten polymorphic micro- and mini-satellite markers, previously designed for the genetic analysis of *T. annulata* (Weir et al., 2007), were used to genotype each *T. annulata* positive sample. Forward primers for each marker set were labeled with a fluorescent dye (FAM) at the 5′ end. Each 25 μl PCR mixture contained 2 μl of template DNA and 1 μl of each forward and reverse primer (10 pmol each). Thermocycler conditions comprised: denaturation at 95 °C for 5 min, 32 cycles at 95 °C for 30 s, 42–62 °C for 30 s and 65 °C for 30 s, followed by a final extension step of 5 min at 65 °C. DNA amplicons were observed on a 2% agarose gel which was pre-stained with ethidium bromide in order to determine the efficacy and specificity of the PCR. PCR products were then sent to Eurofins Ltd. (Eberberg, Germany) where DNA fragment sizes were analysed relative to ROX-labeled GS500 ROX size-standard (Applied Biosystems), using GeneMapper software (Applied Biosystems). This permitted the discrimination of multiple amplicons in a single reaction with a resolution of 1 base pair (bp). Multiple products from a single PCR reaction indicated the presence of several alleles at a locus and therefore a mixture of genotypes. To determine the relative concentration of each allele/amplicon, the area under each peak was assessed. In this way, the predominant allele at each locus was identified for each sample. After testing all ten markers, several were found not to amplify any PCR product, or insufficient product to allow good discrimination of allele sizes. These markers were thus removed from the final analysis, leaving data from the following markers: TS5, TS6, TS15, TS20 and TS25. Good quality data from these five markers was combined to generate a multi-locus genotype (MLG) that represents an estimate of the most abundant *T. annulata* genotype in each sample. As peak area is essentially a continuous variable, it was always possible to identify the most abundant allele in any sample. As *T. annulata* is haploid, the presence of more than one allele at a locus indicates the presence of multiple genotypes. Using the genotyping data, we could therefore calculate an index value representing multiplicity of infection (MOI), i.e. the presence of multiple genotypes per infection. Only minor alleles having a peak height > 20% of the corresponding predominant allele were accepted, in order to eliminate spurious peaks in the vicinity of major peaks, which can artificially inflate the calculated multiplicity of infection (Anderson et al., 2000; Weir et al., 2011). The mean number of alleles across the five high quality loci in each sample was calculated and this index value represented the multiplicity of infection within each sample. The overall mean for the index value for each sample was then calculated to provide the average multiplicity of infection for each host type, i.e. buffalo, native breed cattle and cross breed cattle. Only animals for which data was obtained for at least four out of the five markers was used in the final analysis.

### 2.5 Statistical analyses

#### 2.5.1 Impact of subclinical TBP infection

The haematological parameters of PCV, Hb, and RBC are highly correlated and therefore including them all as independent variables in a statistical analysis can lead to issues of pseudoreplication. Thus, to reduce the number of comparisons performed and to consolidate this clearly correlated data, we reduced the number of variables in the data set in two ways prior to formal analysis. Firstly, a principle component (PC) analysis on our four haematological parameters was performed. This reduced the blood data to two PCs, which cumulatively explained 85% of the variation in the dataset. PC1 was loaded predominantly by three red blood cell-related determinants: PCV (0.379), Hb concentration (0.378) and RBC count (0.356); while PC2 was loaded predominantly by WBC count (0.998). Secondly, among the TBP we detected two principle parasites: *T. annulata* and *Anaplasma* spp. that accounted for the majority of the infections (see Table 1). To reduce the complexity of the dataset and simplify the analyses, we therefore only classified animals with respect to presence of *T. annulata* or *Anaplasma* spp. since we did not have statistical power to determine differences relating to *Babesia bovis* (*n* = 2), or *Babesia bigemina* (*n* = 1) infections. To test whether host breed type influenced subclinical physiological outcomes of infection, we fitted generalised linear mixed models (GLMMs) with the two (blood measure) PCs as response variables. We included host type, sex and age as fixed factors, together with binary data for each of the two different major TBP, and all two- and three-way interactions with TBP and host type. Importantly, we included location (farm of origin) as a random effect in all GLMM analyses. For our purpose, this effectively controls for the non-independence of shared origin for different animals, while allowing us to test for differences in physiological parameters accounting for any potential local variation in these. Where significant interactions were found, we used *post-hoc t*-tests on Least Squares Means (group means adjusted for the significant factors in the model) to determine the groups that differed from one another (see Ponnudurai et al., 2017 for details). All statistical analyses were performed in SAS (v 3.1).

**Table 1:** Differences in TBP infections and haematological parameters in 319 bovines without clinical signs. Counts of different TBP infections (*T. orientalis* not shown as no animals were positive) shown together with Least Squares means and standard error (S.E.) for red blood cell linked parameters PC1, Red Blood Cell counts (RBC), Haemoglobin levels (Hb) and Packed Cell Volume (PCV) in relation to *Theileria annulata* infection status. Adjusted *p*-values (adj p) from least squares means contrasts computed from the full statistical models are provided for each measure and are also shown. Infected native breed cows and buffalo never differ in blood measures from uninfected counterparts. *T. annulata* infected cross-breeds differ significantly in PC1 from uninfected counterparts, probably mediated mostly by differences in Red Blood Cell counts and Packed Cell Volumes.

	*n*	No. of *T. annulata*	No. of *B. bovis*	No. of *B. bigemina*	No. of *Anaplasma* spp.	Mean PC1 uninfected/S.E.	Mean PC1 infected/S.E.	adj p	Mean RBC uninfected/S.E.	Mean RBC infected/S.E.	adj p	Mean Hb uninfected/S.E.	Mean Hb infected/S.E.	adj p	Mean PCV uninfected/S.E.	Mean PCV infected/S.E.	adj p
Cross-breed	113	79	1	0	57	−0.34/0.16	−0.71/0.13	0.03	6.66/0.2	6.05/0.13	0.01	9.8/0.49	9.26/0.39	0.24	29.71/0.87	28.11/0.57	0.12
Native -breed	101	62	1	0	46	0.38/0.15	0.45/0.13	0.67	7.64/0.19	7.51/0.15	0.58	11.50/0.46	11.92/0.42	0.34	33.12/0.81	34.25/0.66	0.27
Water buffalo	103	16	0	1	28	0.34/0.12	0.56/0.22	0.31	6.81/0.13	7.21/0.29	0.21	11.93./0.42	11.76/0.39	0.43	35.39/0.55	36.60/1.26	0.38

#### 2.5.2 Impact of host type on progression to clinical infection

To confirm that host type influenced the likelihood of manifestation of overt clinical signs, we constructed a Generalised Linear Model (GLM) with clinical signs of tick-borne disease (yes or no) as a binary response variable or body temperature as a continuous variable; with Apicomplexa (yes/no), *Anaplasma* spp. (yes/no), sex, age, and host type (native cattle vs crossbreed cattle vs buffalo) as fixed factors. We also used count data representing total number of co-infecting pathogens i.e. when co-infected with two different TBP species a score of two etc. For these analyses, unlike the analyses of physiological responses in subclinical animals above, we chose to combine *T. annulata*, *T. orientalis*, *Babesia bovis* and *Babesia bigemina* infections as one measure: Apicomplexa infection. The animals used for this part of the study from Maharashtra state had a greater proportion of non-*T. annulata* infections than in the sub-clinically infected animals from Tamil Nadu, and we did not wish to exclude these potentially important animals from our analyses, but we lacked a big enough sample size of any individual parasite to analyse alone. While testing individual TBP and specific types of co-infections would have been beneficial, given the low frequency with which they occur it was impossible to do so even with the large sample size (*n* = 233).

#### 2.5.3 Population genetics of *T. annulata*

For the mini– and micro-satellite analyses, Tandem software (Matschiner and Salzburger, 2009) was utilised to facilitate consistent allele-calling. The structure of the *T. annulata* population in different host types was investigated by analysis of molecular variance (AMOVA) (Excoffier et al., 1992). Principal Co-ordinate Analysis (PCoA) was used to visualise the relationship between MLGs using GenAlex6 (Peakall and Smouse, 2006). MOI data was analysed using ANOVA in SPSS, with host type entered as a fixed factor to test for differences in the number of co-infecting parasite genotypes between native breed cattle, cross breed cattle and water buffalo.

## 3 Results

### 3.1 Physiological impact of subclinical TBP infection differs among host types

A brief description of the underlying data used in this analysis is presented in Table 1, showing the differences in TBP prevalence in the 319 animals used for this study, and mean values for blood parameters. A major aim of the present study was to assess whether host type plays a significant role in determining the physiological impact of TBP infections, with a particular emphasis on *T. annulata* infected individuals and with sub-clinically infected animals classified as carriers. The results provide evidence suggesting that host type is indeed a major determinant. PC1, a combined measure that effectively reveals changes in the erythron, was found to be impacted by *T. annulata* infection and host type, the interaction host type*T. annulata status being shown to be statistically significantly associated with differences in PC1 (F_2,305_ = 3.1, *p* = .05). No other interaction was found to be significant (*p* > .334 in all cases) including Apicomplexa*Anaplasma, and so co-infection with the two major TBP had no specific impact on PC1. *Anaplasma* alone status did not impact PC1 (*p* = .34), however we detected an effect of host sex (F_1,305_ = 4.3, *p* = .04) and host age (F_2,305_ = 7.1, *p* < .001). Regardless of host type, males had higher PC1 measures than females, and older animals had lower PC1 measures than younger animals. We used the principle components analysis in place of the individual haematological parameters that compose it, as these are generally tightly correlated and in this way we avoided pseudoreplication. However, we note that treating the different haematological parameters that compose PC1 independently yielded similar results: a reduction in infected crossbreeds compared to uninfected (statistically significant in terms of RBC, and a trend in PCV, see Table 1), and no difference between infected and non-infected native breeds, or water buffalo (see Table 1). While it would have been valuable to assess the impacts of co-infection of other Apicomplexan parasites on health, the small sample size of animals infected with specific pairs of infecting parasite species (*n* = 2) precluded this analysis.

**Fig. 1:**
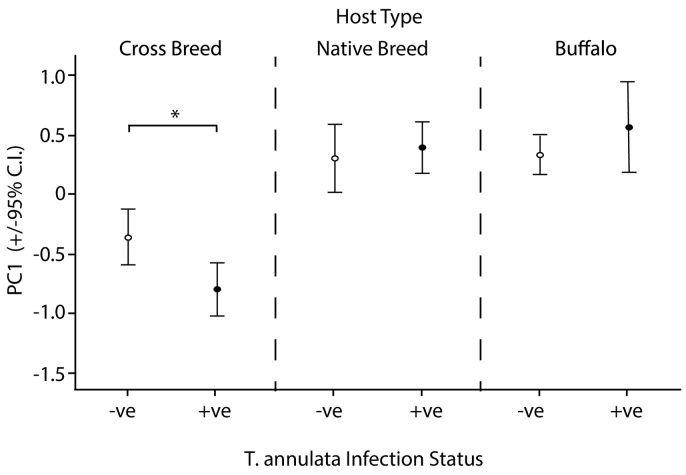
Differences in PC1 by *T. annulata* infection status and host type in subclinically infected carrier animals from Tamil Nadu. Red Blood Cell measurements were significantly lower in *T. annulata*-infected crossbreed cattle than uninfected crossbreed cattle (LSMeans *t* = 2.65, *p* = .0085) but there was no difference between uninfected and infected native breed cattle (*p* = .63) or buffalo (*p* = .45). ^⁎^*p* < .05.

While host types differ in values for PC1 regardless of infection status, there is a clear difference driven by *T. annulata* between infected and uninfected crossbreeds and this is illustrated in Fig. 1. Buffalo and native breeds show no evidence for an infection-mediated difference. Thus, the significantly lower PC1 (with higher values relating to higher scores for RBC, Hb, and PCV) in infected crossbreeds classified as subclinical, carrier state animals, indicates increased susceptibility to establishment/mechanical destruction of parasite-infected RBC and/or the pathological response to infected RBC. There was no such difference for infected, carrier native breed cattle or buffalo, indicating that the costs of TBP infection, in terms of potential destruction of RBC, are exclusively elevated in crossbreed cattle and are primarily due to infection with *T. annulata*. From the raw data, in comparison to *T. annulata*-negative crossbreed cattle, *T annulata*-positive crossbreed cattle were associated with a lower PCV (by 7.4%), Hb concentration (by 8.2%) and RBC count (by 13%) (see Table 1, for statistics and raw data). It is important to note that the animals classified as carrier in this study were diagnosed purely on the basis of PCR: their owners reported no signs of illness preceding or during the period of the study. So although previous clinical disease cannot be discounted entirely it is unlikely that the host breed-mediated response to TBP infection is a result of sampling recently-cleared (or commencing) clinical infections in crossbreed cattle.

The other principle component measure PC2, effectively only illustrates changes in WBC count. For PC2, the greatest impact was for infection by Apicomplexa (F_2,312_ = 5, *p* = .02) regardless of host type (Apicomplexa*host type F_2,310_ = 0.63, *p* = .53). Animals of all host type with carrier Apicomplexa infections had significantly lower WBC counts than animals without carrier infections. There was no statistical impact of the Anaplasma*Apicomplexa interaction term (*p* > .3), so there was no evidence of a specific impact of co-infection on PC2, in concordance with the results for PC1. However, the interaction term Anaplasma*host type was also significant. LSMeans analyses showed that for buffalo and native cattle there was no significant difference in WBC counts between animals infected and uninfected with *Anaplasma* spp. (*p* > .28), but for crossbreed cattle WBC counts were significantly higher in infected animals than uninfected animals (LSMeans *t* = −2.23, *p* = .026), with around a 13% difference between the two. This finding contrasts with the apparent reduction of red blood cells associated with *T. annulata* infections. Higher WBC values in *Anaplasma*-infected crossbreed cattle may reflect an ongoing immune (or stress) response to rickettsia, which is distinct to that of the host response to the other TBP, all of which are species of protozoan.

### 3.2 Progression to clinical disease differs between host types and TBP infections

By comparing the numbers of animals of each main host type displaying clinical signs, with the numbers of each host type in the background population in Maharashtra state, we aimed to determine whether clinical cases were enriched for certain parasites and/or host types. A summary of the underlying data is presented in Table 2. In terms of likelihood of displaying clinical signs associated with disease, there was found to be a significant impact of (a) infection with Apicomplexa, (b) infection with *Anaplasma* spp. and (c) host type. Unsurprisingly, the proportion of infected animals infected with one or more TBPs was greater in the subset of animals displaying clinical signs than in the population as a whole. Regardless of infection status (neither interaction term Apicomplexa*host type or Anaplasma*host type was significant (*p* > .45)), the proportion of native cattle among animals with clinical signs (0.1, *n* = 8/80) was lower than in the population as a whole (0.35, *n* = 54/153). Somewhat surprisingly, the background population was actually enriched for *Anaplasma* spp. infection relative to the clinical cases. This suggests that, in terms of presenting with clinical signs, Apicomplexan species and *Anaplasma* spp. have different impacts, similar to the effect seen on RBC and WBC parameters in the subclinical cases. Interestingly, we found no evidence of any host type specific differences in terms of clinical signs present (*p* > .2 in all cases), including body temperature. This suggests that once Apicomplexan, or at least *T. annulata*, infections manifest as overt clinical disease, host type does not play an important role in determining the outcome of infection, but for cattle at least, native breeds are less likely to progress to this phase than crossbreeds (see Table 2 for numbers of each host type in each analysis).

**Table 2:** Summary of host breed types and TBP counts in animals displaying clinical signs associated with disease vs background population from Maharashtra State, India. The data underlying the statistical models reveals a significant impact of host type (driven by enrichment for crossbreed relative to native breed) as well as Apicomplexan infections in the animals reporting clinical signs. The number of animals infected with pathogens other than *T. annulata* (*T. ann*) and *Anaplasma* spp. (*Ana*) was too small to analyse statistically, but the occurrence of co-infection with *T. orientalis* (*T. or*) is shown. Notably, *Anaplasma* infected animals were not enriched among clinical cases.

	*n*	Cross-breed	Native breed	Water buffalo	Apicomplexa (All)	*T. annulata*	*T. orientalis*	*B. bigemina*	*Anaplasma* spp.	T. ann*Ana	T. ann*T. or	Ana*T. or
Clinical signs	80	42	8	30	31	26	7	4	17	3	3	1
Background population	153	68	54	31	7	7	3	0	92	3	3	0
Total	233	110	62	61	38	33	10	4	109	6	6	1

### 3.3 *T. annulata* population genetics

Animals from both regions we surveyed had more *T. annulata* infections than any other TBP, and the data suggests that this TBP species has the greatest impact on RBC-linked haematological traits, and on the appearance of clinical signs of disease, in different host types. To investigate the question of whether the genotype of this parasite is associated with host type, a subset of *T. annulata* positive samples (*n* = 141) from across our sampling sites were genotyped using a panel of five micro- and mini-satellite markers (Weir et al., 2007). The results from this analysis showed that each of the bovine samples that were positive for *T. annulata* contained multiple genotypes of this parasite. This multiplicity of infection (MOI) is illustrated in terms of the mean number of alleles per locus per isolate, which varied from 1.8 in water buffalo to 3 in cattle (Table 3). However, we found no evidence that the parasite population is sub-structured according to host type or location. Indeed, AMOVA revealed a far higher proportion of the genetic variation was found within (99%) rather than between (1%) host types. Visualisation of the data from the Principal Components analyses highlighted the clear lack of any clustering among host types (Supplementary Figs. S1 and S2). In addition to analysing the *T. annulata* MLG dataset, which is based on the most abundant genotype present in each sample, we also considered the full spectrum of genetic diversity in each sample to assess the MOI. The average number of alleles per locus per isolate was used as a proxy for MOI. Using an ANOVA we observed a significant difference in the MOI between hosts (*p* < .01). This difference was driven by water buffalo for which MOI was lower by an order of 60%: estimated marginal mean number of peaks (± standard deviation) were crossbreed cattle 3.06 ± 0.68; native cattle 3.01 ± 0.59; water buffalo 1.8 ± 0.63.

**Table 3:** Multiplicity of infection in different host types. Mean number of alleles per locus for each of five markers (TS5, TS8, TS15, TS20 and TS25) also showing standard deviation (S.D.), with maximum number of alleles for any marker. Only peaks having peak height > 20% of the corresponding predominant allele were accepted. Data presented as mean across all loci, and mean across all loci with at least one allele present. Water buffalo harboured significantly fewer mixed genotype infections than native and crossbreed cattle.

Host type	*n*	MOI (mean *n* alleles per locus ± S.D.)	MOI (non-0 mean *n* alleles per locus ± S.D.)	Max *n* alleles per locus
Native cattle	37	3.00 ± 0.59	3.02 ± 0.58	8
Crossbreed cattle	71	3.06 ± 0.68	3.08 ± 0.69	6
Water buffalo	33	1.80 ± 0.68	2.00 ± 0.61	5

## Discussion

This study set out to investigate whether the physiological impact of TBP infection differed between host types in terms of clinical signs and haematological parameters classically associated with tick-borne disease. It also assessed whether impact might be associated with preferential infection of different host types by particular sub-populations of the major TBP in India, *T. annulata*. Animals sharing the same habitat were selected for study of subclinical disease impact, in order to control for environmental variation. In addition to evidence that crossbreed cattle are more likely to become clinically affected by TBP, evidence was obtained suggesting that host type impacted infection-linked physiological changes in infected animals not displaying overt clinical signs (i.e. carriers). Apicomplexan-infected, carrier crossbreed cattle had lower packed cell volume, haemoglobin concentrations and red blood cell counts compared to non-infected crossbreed counterparts. In addition, we generated evidence indicating that the lower blood parameters measured in crossbreeds was not associated with detectable differences in the populations of genotypes of *T. annulata* infecting different host types. Thus, the differential susceptibility in response to infection may be viewed as a host-linked physiological trait.

It has long been understood that host genotype can play an important role in mediating the outcome of infection with tick-borne pathogens, especially with regards to manifestation of overt clinical disease. However, it is becoming apparent that sub-clinical effects can be just as economically important, not only in terms of pathogen transmission perpetuating the disease cycle, but also in terms of impact on production-linked traits. Cross-bred *Bos indicus* x *Bos taurus* cattle are kept as a compromise between productivity and disease resistance, and our study confirmed that crossbreeds in our Indian herds lack the disease resistance/infection tolerance of indigenous cattle breeds (Bock et al., 1997, Bock et al., 1999; Oliveira et al., 2008); the proportion of crossbreed cattle in animals with overt clinical disease was greater than native breeds when compared to proportions of each breed type in the population as a whole. However, in terms of animals that did progress to clinical disease, there was no apparent impact of host/breed type in terms of severity of clinical signs. This suggests that, although native cattle are less likely to develop clinical disease than crossbreeds, there were no detectable differences between breed types in the outcome (and potential consequences) of an acute clinical infection. Therefore, as proposed previously, the critical difference between crossbreeds and pure native breeds is likely to be manifest during the early phase of infection, where the macroschizont-infected cell either primes progression towards a protective immune response or the immune pathology of acute disease (Glass et al. 2012).

Importantly, for subclinical infected carriers, the significant reduction in various red blood cell-linked physiological parameters in crossbreed (but not native breed cattle or buffalo) infected with *T. annulata* suggests that the economic cost of subclinical infection will be greater in crossbreeds than the other host types. A detrimental impact of subclinical TBD, including theileriosis (e.g. “Haematocrit disease”) has been reported before (Brown et al., 1990), though to our knowledge this is the first confirmation that host type mediates these impacts in apparently healthy carrier animals (while controlling for shared origin) in India or elsewhere.

In our study we analysed each animal at only a single time-point; this precludes an estimate of the impact of sub-clinical infection on milk production itself, which requires monitoring over the course of a lactation. However, though the reduction in blood parameter values we measured may seem relatively modest (between 7.4 and 13%) these are still of potential impact on productivity. A recent study of *T. orientalis* in Australia showed a reduction in PCV of just 5% was associated with a decrease in milk fat (−13 kg) and milk protein (−8 kg) after 100 days, and losses of around 115 l of total milk after 305 days (Perera et al., 2014). These parameters may be especially important, as the price at which farmers can sell milk varies by fat content and, together with potential role of carriers in onward transmission to susceptible animals, should be taken into account when determining the true impact of TBD. The results of our study, therefore, indicate that while conventional cross-breeding programmes in India do result in more disease resistant herds, the ability to obtain maximum economic output is constrained by TBP infection, and there is a need for accurate assessment of the full economic cost of TBP carrier infection in cross-bred animals. Moreover, if greater economic output of ruminants in countries endemic for TBP is to be achieved then investigation of the mechanisms that prevent progression to clinical disease and promote resistance to the subclinical impacts of infection is highly relevant. It also raises the question of whether, in certain circumstances, the use of transmission-blocking methods, which offer the prospect of maintaining TBP-free livestock, have a place among the currently used disease control modalities.

The results from our mini- and micro-satellite analysis suggest that the differences we observed in host physiological changes mediated by infection (at least for *T. annulata*) are not driven by different parasite genotypes infecting cows. Like the situation previously documented for *Bos taurus* cattle in other endemic regions (Weir et al., 2011), the parasite population appears diverse, in general, and there is no evidence of over-representation of a single, virulent genotype or sub-structuring of the population with different host types. Thus, the samples we analysed showed a great diversity of parasites genotypes across all three host types, with much more diversity within than between host types. This indicates that co-crazed hosts of different types are infected with similar parasite populations, and that there is no preference for different *T. annulata* genotypes to establish infection in any of the bovine hosts studied. Despite this high level of diversity, water buffalo had significantly lower MOI than either of the cattle host types. This result suggests that buffalo are probably infected by the same parasites as cattle, but have fewer circulating genotypes. This difference could be generated by several potential mechanisms, and may be explained by: 1. greater resistance to becoming infected with multiple genotypes of *T. annulata* than cattle; 2. suppression of parasitaemia to below the level maintained in cattle, reducing the ability to detect and quantify different genotypes; 3. increased ability to clear certain genotypes of *T. annulata,* before reinfection with new genotypes; 4. increased immunity to reinfection with divergent parasite genotypes; 5. increased competition between *T. annulata* genotypes within infected buffalo hosts. It is not within the scope of this study to elucidate the precise mechanism, but regardless of how this phenomenon arises, our results indicate an epidemiological context for *T. annulata* in Indian bovine hosts that is quite distinct from *T. parva* in African bovine hosts. In *T. parva*, cape buffalo have been reported to harbour a greater MOI and diversity of parasite genotypes than cattle, and thus can act as a reservoir of infection for susceptible dairy cattle, with only a small subset of genotypes actually associated with theileriosis in cattle (Bishop et al., 1994; Collins and Allsopp, 1999; Geysen et al., 2004; Oura et al., 2003). However, a recent study suggests some of this effect may be explained by loss of genotypes through rapid death of naïve infected cows, rather than failure to transmit into the bloodstream (Sitt et al. 2019). Previously, it has been shown that water buffalo are less likely to display carrier infections than either native or crossbred cattle in India (Ponnudurai et al., 2017; Kolte et al., 2017), and are less likely to test positive by microscopy (in comparison to PCR), suggesting that they show greater resistance to becoming infected or developing a detectable parasitaemia. The observed difference in epidemiological context between *T. annulata* and *T. parva* may therefore reflect a distinct evolutionary history that has generated divergence in resistance and tolerance efficacies between different indigenous bovine and *Theileria* species.

## Ethical statement

This study was carried out in strict accordance with the recommendations of the Veterinary Council of India and all field work was overseen by their staff. Ethical approval was not required by the ethical committee for performing animal experiments (Institutional Animal Ethics Committee of Nagpur Veterinary College and Institutional Animal Ethics Committee of Tamil Nadu Veterinary and Animal Sciences University) as: 1. the survey was conducted by government official veterinary physicians in village farms within their jurisdiction, 2. samples were taken as part of the standard course of veterinary inspection to determine presence of infections detrimental to animal health in India and 3. in India, ethical approval is not required for survey work conducted for the benefit of livestock welfare or improved farming practice, as in this case. No animals were housed or harmed as part of the survey, and every care was taken while restraining animals in order to collect samples.

## Competing interests

The authors have no competing interests to declare.

## Appendix A Supplementary data

Supplementary material Image 1
