# Supplementary material for: The impact of tick-borne pathogen infection in Indian bovines is determined by host type but not the genotype of Theileria annulata
Source: Infect Genet Evol. 2019 Nov;75:103972. doi: 10.1016/j.meegid.2019.103972 (PMC6853275; doi:10.1016/j.meegid.2019.103972)
Supplement: Supplementary file 1 — Supplementary material [file mmc1.docx]

**Supplementary Figure S1: Principle Components Analysis of *Theileria annulata* multi-locus genotypes based on panel of five markers labelled by (a) host type and (b). location.** In both cases the plots are clearly showing that there is no evidence of clustering of genotypes by either host type or sampling location. This suggests the parasite population is not sub-structured by host type or by geography and may represent a single underlying population.

**a.**

**b.**
